# Sequential Single Shot X-ray Photon Correlation Spectroscopy at the SACLA Free Electron Laser

**DOI:** 10.1038/srep17193

**Published:** 2015-11-27

**Authors:** Felix Lehmkühler, Paweł Kwaśniewski, Wojciech Roseker, Birgit Fischer, Martin A. Schroer, Kensuke Tono, Tetsuo Katayama, Michael Sprung, Marcin Sikorski, Sanghoon Song, James Glownia, Matthieu Chollet, Silke Nelson, Aymeric Robert, Christian Gutt, Makina Yabashi, Tetsuya Ishikawa, Gerhard Grübel

**Affiliations:** 1 Deutsches Elektronen-Synchrotron DESY, Notkestr. 85, 22607 Hamburg, Germany; 2 The Hamburg Centre of Ultrafast Imaging, Luruper Chaussee 149, 22761 Hamburg, Germany; 3 Japan Synchrotron Radiation Research Institute, 1-1-1 Kuoto, Sayo-cho, Sayo-gun, Hyogo 679-5198, Japan; 4 Linac Coherent Light Source, SLAC National Accelerator Laboratory, 2575 Sand Hill road, Menlo Park, CA 94025, USA; 5 Department Physik, Universität Siegen, Walter-Flex-Str. 3, 57072 Siegen, Germany; 6 RIKEN SPring-8 Center, 1-1-1 Kuoto, Sayo-cho, Sayo-gun, Hyogo 679-5148, Japan

## Abstract

Hard X-ray free electron lasers allow for the first time to access dynamics of condensed matter samples ranging from femtoseconds to several hundred seconds. In particular, the exceptional large transverse coherence of the X-ray pulses and the high time-averaged flux promises to reach time and length scales that have not been accessible up to now with storage ring based sources. However, due to the fluctuations originating from the stochastic nature of the self-amplified spontaneous emission (SASE) process the application of well established techniques such as X-ray photon correlation spectroscopy (XPCS) is challenging. Here we demonstrate a single-shot based sequential XPCS study on a colloidal suspension with a relaxation time comparable to the SACLA free-electron laser pulse repetition rate. High quality correlation functions could be extracted without any indications for sample damage. This opens the way for systematic sequential XPCS experiments at FEL sources.

Studying dynamics of complex materials is one of the key topics at X-ray free electron laser (FEL) facilities. In particular, dynamics ranging from femtoseconds to seconds over length scales between atomic dimensions and several hundred nm can be studied by X-ray Photon Correlation Spectroscopy (XPCS)^1^,^2^,^3^. XPCS is a well established technique to probe the time evolution of condensed matter systems using coherent X-rays, covering, e.g., magnetic domain fluctuations^4^, rheological properties of soft matter^5^, capillary wave flucutations^6^, and slow dynamics in glass-forming systems^7^,^8^,^9^. A more detailed overview on XPCS can be found in recent review publications^10^,^11^,^12^.

In conventional XPCS, so-called sequential-mode XPCS, the temporal evolution of the sample is obtained by tracking changes of the speckle patterns over time. For ultrafast dynamics in the fs to ns regime, split-pulse techniques have been proposed and are currently under commissioning or development at hard X-ray FEL facilities^13^,^14^,^15^,^16^,^17^,^18^.

The accessible time scale of sequential XPCS is determined by the repetition rate of the linac at FEL facilities. Currently this allows studies down to 8.3 ms at the Linac Coherent Light Source (LCLS) at the SLAC National Accelerator Laboratory (USA) or 16.6 ms at the Spring-8 Angstrom Compact free-electron Laser SACLA (Japan)^19^,^20^,^21^. With the European XFEL, the time resolution will be strongly reduced to the 10^−7^ s regime.

Hard X-ray FEL sources are excellent machines to perform sequential XPCS experiments. First, FEL beams exhibit a superior degree of transverse coherence compared to storage ring based sources; more than 80% are easily achieved^22^,^23^,^24^,^25^,^26^. In general, this allows using larger beams and thus potentially less sample damage. Second, current FEL sources provide a photon flux of 10^11^–10^12^ coherent photons per single shot in pink beam mode and about 10^9^–10^10^ monochromatized photons. In contrast, state-of-the-art storage ring beamlines are limited to about 10^9^–10^11^ photons per second depending on the chosen setup. Thus, FEL sources still provide at least a factor of 10 more coherent photons, allowing to access a factor of 100 shorter relaxation times in XPCS experiments^12^. Future sources such as the European XFEL will provide 10^13^ up to 10^14^ photons per pulse, enabling sequential XPCS at time scales to the sub-μs regime. Third, using a pulsed source such as an XFEL, thermal effects to the sample might be reduced. As discussed by Grübel *et al.*^2^ the pulsed structure of the incoming beam allows thermal relaxation of the sample between two subsequent pulses. This is typically not possible at storage ring sources, where the incoming flux has to be reduced in case of radiation sensitive samples. This will be of big benefit to a large class of experiments.

In particular, dynamics studies at large scattering vectors, i.e. probing molecular and atomic length scales^27^,^28^,^29^,^30^ are challenging at storage ring sources due to the small coherence lengths and low photon flux. For such weakly scattering sample systems, the accessible time scale would be reduced from several seconds^27^,^29^ to the millisecond regime and below for future XFEL sources.

Sequential XPCS experiments, however, strongly rely on stable beam conditions, e.g. intensity, contrast and pointing beam stability. While these demands are fullfilled at storage rings, the radiation generated in FEL machines can be dominated by various fluctuations. Therefore, the influence of shot-to-shot fluctuations originating from the stochastic nature of the Self-Amplified Spontaneous Emission (SASE) process in current hard X-ray FELs has to be characterized before performing sequential XPCS.

Carnis *et al.*^31^ reported recently a sequential XPCS study on slow dynamics of gold nanoparticles in a molten polymer at the LCLS. In their work, the slow detector read-out of seven seconds restricted the study to slow samples with relaxation times of several 100 seconds. In addition, due to the very weakly scattering sample, it was necessary to integrate over 100 x-ray pulses in order to overcome intensity limitations and shot-to-shot fluctuations. The averaging increased the signal-to-noise ratio, allowing the reach of higher *q* values at the cost of low contrast (*β*^2^ ≈ 0.08–0.12). This low contrast was interpreted as a consequence of SASE pointing fluctuations. Whether or not this is a general phenomenon to be encountered at XFEL machines is of outmost importance for the applicability of sequential mode XPCS. This issue is addressed in this paper.

In XPCS experiments, the sample dynamics is resolved using the correlation function of the scattered intensity *I*(*q*, *t*), given by


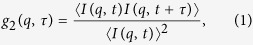


where *τ* is the lag time (in the case of pulsed FEL sources defined by the repetition rate), 〈…〉 denotes an ensemble average over all equivalent times *t* and detector pixels contained in a chosen wave vector range *q* ± Δ*q*, *q* being the modulus of the scattering vector. The measured intensity autocorrelation function can be related to the intermediate scattering function *g*_1_(*q*, *τ*), assuming that the temporal intensity fluctuations are well described by a normal distribution, using the Siegert relation


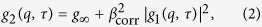


where *β*_corr_ is the speckle contrast, and *g*_∞_ is the baseline (equal to 1 for ergodic samples). In the simple case of the diffusive motion of spherical particles, *g*_1_ is a simple exponential decay *g*_1_(*q*, *τ*) = exp(−Γ(*q*)*τ*) with Γ(*q*) = *Dq*^2^. *D* is the translational diffusion coefficient. Obviously, fluctuations of contrast or beam intensity will have significant influence on the extraction of *g*_2_ functions.

Here we describe a demonstration experiment at SACLA on a prototypical complex fluid showing that single XFEL pulses can be used to measure the equilibrium dynamics of dilute colloidal suspensions by sequential XPCS. The sample was chosen so that its dynamics can only be accessed by sequential shots of the SACLA linac. Correlation functions *g*_2_ with sufficient statistics could be extracted for as few as 50 single shots of an average length of 5.2 fs corresponding to a total X-ray exposure of 260 fs. A detailed analysis shows that there is also a reduced speckle contrast which is approximately a factor of 2 below the expected value defined by the coherence properties of SACLA^25^. The experiment was however carried out in pink beam mode (without monochromator) so that the reduced temporal coherence also has to be considered. The contributions of eventual beam instabilities was investigated in detail. The results clearly demonstrate the feasibility of sequential single shot XPCS experiments at FEL sources without sample damage.

## Results

**Figure 1:**
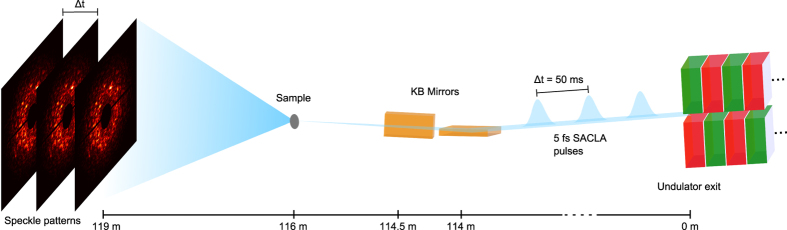
Schematic setup of the experiment. Each pulse gives rise to a speckle pattern used for the XPCS analysis.

**Figure 2:**
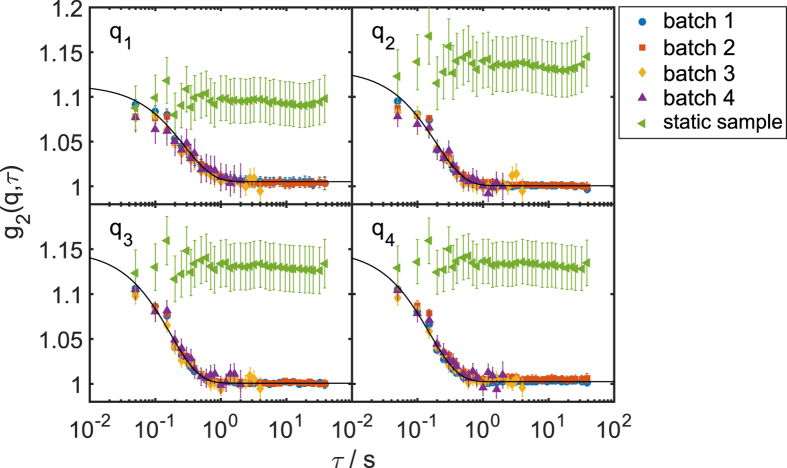
Correlation functions from different runs explained in the text. They are fitted with the simple exponential decay (solid black line) for different *q* values: *q*_1_ = 0.025 nm^−1^, *q*_2_ = 0.028 nm^−1^, *q*_3_ = 0.031 nm^−1^, and *q*_4_ = 0.034 nm^−1^. Data from the static sample are plotted with the green triangles.

Single shot speckle patterns were taken from both a static sample consisting of PMMA spheres and a dynamic sample consisting of silica spheres dispersed in glycerol (see Methods for details). An overview of the experimental setup including all key components is shown in Fig. 1. The patterns were analyzed on a single-shot basis and *g*_2_-functions were extracted for different batches of subsequent patterns. In Fig. 2 the results are shown for both samples at four selected *q*-values. As expected, the static sample does not show any time dependence, i.e., the *g*_2_-function stays constant. In order to check for radiation effects on the measured dynamics of the silica sample, four batches of data were chosen from the complete, 10^4^ frame long, set of speckle patterns, collected at the same sample position. With the average pulse length of 5.2 fs^25^,^32^, this results in a total exposure time of 52 ps. For batch 1, the first 1000 shots, i.e. 5.2 ps total exposure, were analyzed, while batch 2 consists of 1000 shots from the end of the data set. To evaluate the effect of statistics, batch 3 comprises the first 100 shots and batch 4 further 50 shots from the middle of the data set. Details of the various batches are summarized in Table 1. The case of batch 4 corresponds to a total exposure time of approximately 260 fs. Deviations of the correlation functions obtained from these data would show whether the colloidal dynamics changes (e.g. due to radiation damage) during data acquisition. As can be seen from Fig. 2, all correlation functions overlay, indicating no influence from X-ray damage to the sample dynamics. Furthermore, the results from batches 3 and 4 do not differ significantely from the other batches except for larger error bars. This proves that the short runs of 50–100 shots, i.e. 2.5–5 s total experimental time at 20 Hz repetition, is sufficient to measure the sample dynamics.

**Figure 3:**
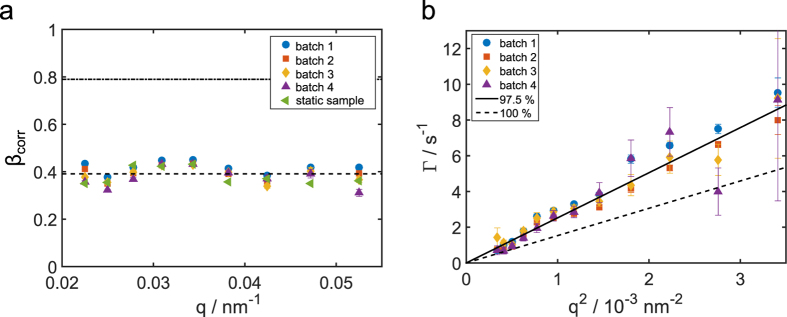
(**a**) The fitted value of contrast *β* as a function of *q* for all runs. The dashed line represents the averaged value of 〈*β*_corr_〉 = 0.39, the dashed dotted line the single shot value *β*_*s*_ = 0.79 from^25^. (**b**) Relaxation rate Γ as a function of *q*^2^ for all runs together with a model assuming 97.5% (solid line) and 100% (dashed line) glycerol, respectively.

The *g*_2_-functions were fitted with a single exponential decay


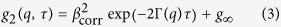


with the relaxation rate Γ(*q*) = 1/*τ*_*c*_(*q*), where *τ*_*c*_ denotes the characteristic relaxation time of the sample. In general, the contrast *β*_corr_ should equal the degree of coherence *β*_s_ of the X-ray beam^33^,^34^,^35^,^36^ if parameters such as detector resolution and speckle size can be neglected. Since the full unmonochromatized SASE beam with a bandwidth of 0.5% was used in this experiment, the measured contrast of the speckle pattern *β*_s_ is affected by the limited longitudinal coherence length. This can be corrected by a *q*-dependent correction factor (see methods) following refs 35,36 as shown in our previous publications^23^,^24^,^25^. At SACLA, we found a single shot contrast of *β*_single_ = 0.7 ± 0.1, yielding a transverse coherence of 0.79 ± 0.09 after correction for the longitudinal coherence length. From the *g*_2_-functions of the dynamic samples 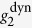, the contrast can be obtained in the *τ* → 0 limit. The correlation function 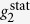 from the speckle patterns of a static sample shows no time dependence. Since this sample does not show any dynamics, one obtains the constant value 
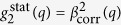
 for all lag times *τ*. The fit of the *g*_2_-functions for the dynamic sample yielded contrast values similar to the ones obtained from the static sample. The results that were corrected for contribution from the longitudinal coherence length are shown in Fig. 3(a). The contrast values are given in Table 1. We found a *q*-independent average contrast of 〈*β*_corr_〉_*q*_ = 0.39 ± 0.03. This is about a factor of 2 below the average single shot contrast^25^.

A plot of the relaxation rate Γ as a function of *q*^2^ is presented in Fig. 3(b). In this representation, diffusive motion of the particles manifests itself as a linear dependence of Γ ∝ *q*^2^, with the slope corresponding to the diffusion coefficient *D*. This was calculated by taking the fixed temperature in the experimental hutch (*T* = 27 °C) into account, the measured particle radius (*R* = 188 nm) and the mass concentration of glycerol *C*_*m*_. The result for 100% glycerol is shown by the dashed line, clearly underestimating the experimental results. This discrepancy could arise underestimating the sample temperature or overestimating the viscosity of the solvent. Since we were able to obtain *g*_2_ functions and observed a quadratic relation Γ ∝ *q*^2^, direct beam damage of the particles can be neglected. In order to estimate the impact from thermal effects on the sample, the photon energy for each pulse was measured by a calibrated photodiode placed upstream from the KB mirrors. We estimate that an average of approximately 2.3 ⋅ 10^4^ photons per pulse was absorbed by a silica sphere (see Methods for more details). This corresponds to a heat increase of about Δ*T*_si_ ≈ 500 K/unit which is far below the melting point of silica around 2000 K. The same calculation for glycerol results in a moderate increase of Δ*T*_gl_ ≈ 37 K. The deposited energy relaxes by thermal diffusion^2^,^37^ on the time scale of *t*_th,gly_ = 10 μs for glycerol and *t*_th,gly_ ≈ 1 μs for silica. We are thus forced to conclude that beam-induced steady heating can be neglected because of the repetition rate of 20 Hz, which ensures the thermal relaxation of the sample between subsequent pulses. An appropriate description of the data can be achieved by allowing a minute water content in the glycerol solvent. Assuming a glycerol mass concentration of *C*_*m*_ = 0.975 ± 0.002, which reflects a small water content due to the hygroscopic nature of glycerol, we obtain the solid line in Fig. 3(b), matching the experimental data.

**Figure 4:**
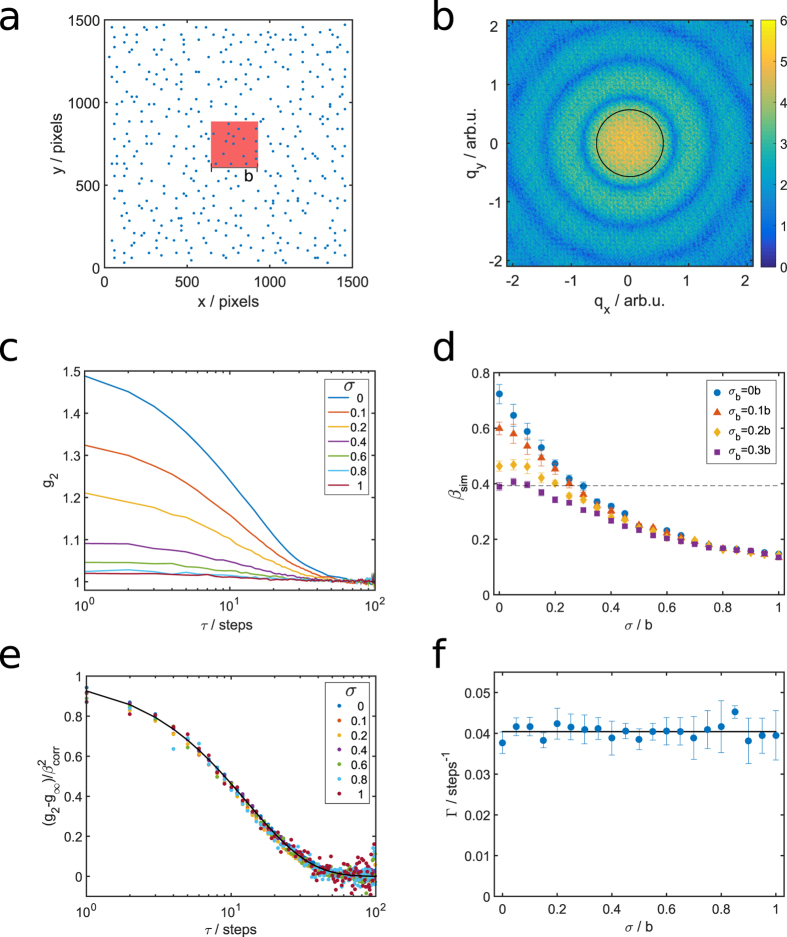
Simulation of beam movement. (**a**) Snapshot of a simulated sample. The red square is a typical cut out representing the X-ray beam. (**b**) Typical speckle pattern calculated from (**a**). The intensity is given in arbitrary log-scale units indicated by the colorbar. (**c**) Correlation functions *g*_2_ calculated from the simulated scattering patterns for a fixed *q* at different random translations with standard deviation *σ*. (**d**) Contrast *β*_sim_ as function of *σ* in units of the beam size *b* for four different beam size modulations *σ*_*b*_. The error bars were estimated by repeating the simulations 24 times. The dashed line represents the experimental value of *β*_corr_ = 0.39. (**e**) Correlation functions *g*_2_ normalized on the contrast 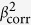 for different values of *σ* (*σ*_*b*_ = 0). (**f**) Extracted relaxation rates for different *σ* (*σ*_*b*_ = 0).

In order to estimate the influence of spatial shot-to-shot fluctuations^38^, we performed 2D simulations of diffusing spherical particles with an additional random beam displacement and beam size fluctuations (see methods for details). A sketch of the simulation box is shown in Fig. 4(a), and a representative calculated speckle pattern from this arrangement can be found in Fig. 4(b). First, we extracted *g*_2_-functions for different degrees of simulated beam displacements with standard deviation *σ* at a fixed *q*. The results are shown in Fig. 4(c). The dominant effect on the *g*_2_-functions is a decreasing amplitude which is reflected in a loss of contrast *β* as function of the increasing amplitude of beam displacement (see Fig. 4(d)). Nevertheless, the extracted relaxation rate Γ does not differ within the error bars. Most importantly, as long as the beam displacement is random, no further dynamics contribution is observed. This is demonstrated by normalized correlation functions in Fig. 4(e) and the extracted relaxation rates in Fig. 4(f). Second, an additional change of beam size was modeled with a standard deviation *σ*_*b*_. The resulting contrast *β*_sim_ as function of *σ* at selected beamsize modulations *σ*_*b*_ is shown in Fig. 4(d). Without beam displacement and stable beam size (i.e. *σ* = 0, *σ*_*b*_ = 0), we observe a contrast of *β*_sim_(*σ* = 0) = 0.72 ± 0.03, which is already slightly below the input value of the spatial coherence of *β*_s_ = 0.79 ± 0.09, and drops with increasing *σ*. The experimentally obtained value of *β*_corr_ = 0.39 ≈ 0.5*β*_s_ is achieved for *σ* ≈ 0.28*b*. This corresponds to a mean displacement amplitude of less than 0.5 μm. Taking additional beam size changes into account, a contrast drop to 0.39 can be observed for *σ* = 0.2*b* and *σ*_*b*_ = 0.2*b*. However, we did not take further shot-to-shot variations into account, e.g., jittering incident intensity or change of the beam shape. Such contributions could further lower the contrast.

## Discussion

We demonstrated that single-shot sequential XPCS can be performed at a hard X-ray FEL. Using a prototypical soft matter sample we were able to measure the diffusion of silica spheres in glycerol with a total exposure time of approximately 260 fs. The extracted relaxation rates were modeled by a glycerol-water mixture model with a glycerol fraction of 0.975. Most importantly, there were no indications of direct beam damage during 10000 shots illuminating the sample. The impact from beam-induced heating could be neglected because of the fast timescale of thermal diffusion compared to the repetition rate of SACLA. It is important to note, that such thermal effects will be of major importance at future FEL sources where the distance between two pulses is similar to or shorter than the time scale of thermal diffusion. The contrast obtained from the *g*_2_-functions was found to be approximately a factor of two below the single-shot contrast. In general, such a drop of contrast can originate from fast and unresolved processes or beam damage. However, there was no evidence for neither fast processes because the static sample shows the same contrast as the dynamic one nor beam damage as the results for batches taken at the beginning and end of the complete data sets do not differ. We were able to model this reduction of contrast by simulations of a moving and size-changing beam, as observed at FEL facilities^38^,^39^. The stability of the beam at SACLA has been previously found to have very small Gaussian distributed beam displacement amplitudes of the full SASE beam with standard deviation *σ*^exp^ ≈ 0.1*b* and beam size stability of 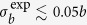 upstream the experimental hutch^38^. With these values, our simulation would yield a contrast of 0.55. This value is still above the experimentally obtained contrast from the *g*_2_-functions of 0.39 ± 0.03. It is however important to mention, that the stability measurements were performed upstream of the Kirkpatrik-Baez (KB) mirror system and high resolution data from the focused beam at the sample position are not available. Whether or not the difference can be attributed to slightly larger fluctuation at the sample position and other shot-to-shot wavefront distortions^39^ is presently not clear.

In conclusion, we demonstrated a single-shot based sequential XPCS experiment on silica spheres at the FEL SACLA. The results reflect the diffusive nature of the spheres, indicating no beam damage of the sample. The lower contrast of the *g*_2_-function was modeled by fluctuations of beam size, pointing stability and coherence variations. Our results prove the feasibility of sequential XPCS at FEL sources and pave the way to experiments at upcoming facilities such as the European XFEL allowing to access timescales below microseconds.

## Materials and Methods

### Sample preparation

The static sample was prepared by drying a suspension of Poly(methyl-methacrylate) (PMMA) colloidal spheres in decalin inside a quartz capillary. The particles have a mean radius of *R* = 125.5 nm with a polydispersity of 7.0% that was determined by fitting the particle form factor extracted from scattering patterns from diluted suspensions.

The dynamic sample is composed of 188 nm radius spherical silica particles with a size polydispersity of 3.5% suspended in glycerol. The particles were initially prepared in ethanol using a modified Stöber method^40^,^41^. After the synthesis the silica particles were dialysed against deionized water. Afterwards the solvent was exchanged by adding gylcerol to the original suspension and removing the water in a rotary evaporator. The volume fraction of the silica particles was set to *ϕ* ≈ 0.01. At this volume fraction, no structure factor *S*(*q*) can be measured, confirming the Brownian motion nature of the particles. Prior to transferring to a quartz capillary for the measurement, the sample was kept in an oven under vacuum at 50 °C to prevent water absorption. The capillaries were vacuum sealed afterwards. The particle size and the sample dynamics were pre-characterized at beamline P10 of PETRA III at DESY, Germany.

### Experimental configuration

The experiment was performed at EH3 of BL3 beamline of the Spring-8 Angstrom Compact Free Electron Laser (SACLA)^38^ in a small-angle X-ray scattering (SAXS) geometry. The photon energy was set to 8 keV with a bandwidth of Δ*E*/*E* ~ 5 × 10^−3^. X-ray pulses of (300 ± 40) μJ energy were produced at a repetition rate of 20 Hz. The beam was focused with KB mirrors to 1.8 × 1.5 μm^2^ at the sample position as measured by knife edge scans. The samples were placed in the MAXIC chamber^42^. The detector (dual multiport charge coupled device (MPCCD))^43^ was located at a sample-detector distance of 3 m. At least 10000 single shot diffraction patterns were taken for each sample.

## Data analysis

Intensity correlation functions were calculated using the multi-tau algorithm^44^. Normalization of the scattering data to the azimuthal average within the same frame enabled the measurement of static speckle contrast (see green triangles in Fig. 2).

### Impact from longitudinal coherence

The impact of the effect of the longitudinal coherence to the speckle contrast can be calculated to^23^,^24^,^25^,^35^,^36^


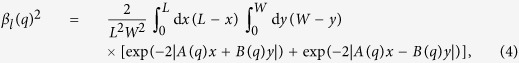


with beam size *L*, sample thickness *W*, and the coefficients 
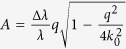
, 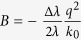, and *k*_0_ = 2*π*/*λ*. In SAXS geometry, this factor is typically equal to 1 for bandwidth of monochromatized beams (Δ*λ*/*λ* ≤ 10^−4^). Here we used the natural bandwidth of the FEL radiation of Δ*λ*/*λ* = 5 × 10^−3^, resulting in correction factors around 0.9^25^.

### Beam-assisted heating of the sample

Knowing the transmission of the focusing system (*Tr*_KB_ = 0.94)^45^ and of the attenuators used to prevent the detector from being overexposed, we can estimate the energy delivered to the sample position. For the dynamic sample measurement this was on average 0.31 μJ per pulse, which corresponds to 2.4 × 10^8^ photons/pulse. Dividing this value by the beam area gives an average photon density of 8.9 × 10^15^ photons/pulse/cm^2^ which is comparable to or lower to previous experiments at LCLS that did not see either any indication of beam damage^23^,^31^,^37^. Using the absorption cross section of the silica particles, a simple calculation yields an average of 2.3 ⋅ 10^4^ photons per pulse absorbed by a silica sphere. This corresponds to 2.5 eV per SiO_2_ unit. Assuming that this energy is completely converted to heat and stays within the particles, we obtain a heat increase of Δ*T*_Si_ ≈ 500 K/unit. A similar calculation yields a moderate increase of Δ*T*_gl_ ≈ 37 K for glycerol. The deposited energy relaxes by thermal diffusion whose time is given by *t*_th_ = *d*^2^/(4*D*_th_), where *D*_th_ is the thermal diffusivity^2^ and *d* the relaxation length scale. Here, we used *d* = 2 μm which is slightly larger than the beam size and literature values of the thermal diffusivity^46^.

### Viscosity of glycerol-water mixtures

Although much care was taken to prevent water absorption, it is likely that the sample contained a small amount of water due to the hygroscopic character of glycerol. Since the water content strongly influences the viscosity *η* of the mixture^47^,^48^, it is important to take this factor into account when measuring the diffusion coefficient which depends on *η* according to the Stokes-Einstein relation


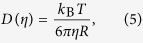


where *k*_B_ is the Boltzmann’s constant, *T* is the temperature and *R* is the particle radius.

Cheng^49^ proposed an empirical formula, expressing the viscosity of glycerol/water mixture as a function of two variables: the mass concentration of glycerol 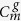 and temperature *T*. The viscosity *η* of a mixture is related to those of its components, water (*η*_*w*_) and glycerol (*η*_*g*_), as


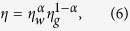


where *α* varies as a function of 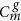 as


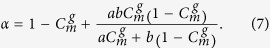


The temperature dependence of the coefficients *a* and *b* and the viscosities *η*_*w*_ and *η*_*g*_ are determined empirically and chosen to comply with experimental data^49^.

## Pointing fluctuations

In order to model the influence of spatial instabilities of the X-ray beam, we performed two-dimensional simulations of diffusing spherical particles. 400 spherical particles were placed on lattice points in a 1500 × 1500 pixels grid. To obtain a disordered sample, a sequence of random movements was performed. In each step, the Voronoi tesselation was calculated for the system and every single particle was moved randomly inside its Voronoi cell. After ten repetitions no positional order is present, see Fig. 4(a). The area fraction of the particles was about 0.012.

The degree of coherence of the incoming beam was set to *β*_s_ = 0.79 ± 0.09, following our previous results^25^. For each simulation step of Brownian motion, every particle moves independently by a randomly oriented step following a Gaussian distribution of step lengths. Beam displacements were simulated from a stable beam (*σ* = 0) up to *σ* = *b*, where *b* denotes the beamsize. Speckle patterns were calculated after each step from a region of interest representing a square sized beam with size *b*, see Fig. 4(a). Beam movement was modeled by moving the region of interest by Gaussian distributed step lengths in *x* and *y*-direction for each simulations step, respectively. Analogously, beam size changes were simulated by changing the size of the region of interest by a Gaussian distributed value in *x* and *y*-direction for each simulations step, respectively. These distributions resemble the beam stability found experimentally upstream the KB system^38^. Afterwards, *g*_2_-functions were extracted. This was repeated for different standard deviations *σ* of the Gaussian distribution function, for each *σ* the simulation was repeated 24 times to obtain a reliable statistics. Other distributions, e.g., exponentially or Gamma-distributed moving vectors, resulted in similar results, with slightly shifted contrast values.

## Additional Information

**How to cite this article**: Lehmkühler, F. *et al.* Sequential Single Shot X-ray Photon Correlation Spectroscopy at the SACLA Free Electron Laser. *Sci. Rep.* **5**, 17193; doi: 10.1038/srep17193 (2015).

## Figures and Tables

**Table 1:** Overview of batch details and resulting contrast *β*_corr_.

Batch	No. of shots	Pos. in run	*β*_corr_
1	1000	start	0.42 ± 0.03
2	1000	end	0.39 ± 0.03
3	100	start	0.38 ± 0.03
4	50	middle	0.38 ± 0.04
static	1000	start	0.39 ± 0.04
